# Offering Tailored Therapy for Patients with Benign Esophageal Strictures—A Tertiary Center Experience in Romania

**DOI:** 10.3390/jcm14072181

**Published:** 2025-03-22

**Authors:** Gheorghe G. Balan, Elena Toader, Sebastian Zenovia, Simona Juncu, Andreea Iacob, Robert Nastasa, Catalin Victor Sfarti, Anca Trifan, Anton Knieling

**Affiliations:** 1 Institute of Gastroenterology and Hepatology, St. Spiridon Iasi County Emergency Clinical Hospital, 700111 Iasi, Romania; 2 General Medicine Department, Grigore T. Popa University of Medicine and Pharmacy of Iasi, 700115 Iasi, Romania; anton.knieling@umfiasi.ro; 3 Institute of Legal Medicine, 700455 Iasi, Romania

**Keywords:** balloon dilation, esophageal stenosis, dysphagia, peptic stricture

## Abstract

**Background**: Over the last two decades, therapy for benign esophageal strictures has shifted from empirical dilatations and surgery to evidence-based and complex endoscopic and surgical procedures, aiming to achieve long-term esophageal patency. Aim: The purpose of our study is to provide descriptive evidence regarding the appropriate tailored medical, endoscopic, and surgical management of benign esophageal strictures. **Methods**: This retrospective study includes patients with benign esophageal strictures; the data collected encompass the complete patient profiles, detailed etiologic and anatomic workups of the strictures, comprehensive imaging, as well as management and follow-up details. Technical and clinical success rates, adverse events, stricture patency, and the need for additional therapy have been evaluated. **Results**: Most of the strictures (80.2%) were complex, requiring advanced techniques for management. The primary treatment involved endoscopic dilation, performed with Savary-Gillard bougie dilators in 76.7% of cases and pneumatic balloon dilators in 23.3% of cases. Clinical success was achieved in 95.3% of patients, with a significant improvement in the Ogilvie dysphagia score. Patients with caustic strictures required repeated dilations over the years, compared to shorter intervals for peptic strictures. Adverse events were minimal (e.g., perforation 2.3% and bleeding 4.7%) and managed predominantly endoscopically. Refractory strictures (16.3%) required advanced interventions, including fully covered self-expandable metallic stents (fc-SEMS) and corticosteroid injections. **Conclusions**: Both our data and the current literature support the use of tailored endoscopic strategies as the first-choice options for managing benign esophageal strictures. Our results strongly suggest against one-size-fits-all therapeutic alternatives.

## 1. Introduction

There are numerous causes of benign esophageal strictures (BESs). The most frequent are peptic strictures, esophagitis, radiotherapy-induced esophagitis, Schatzki rings, surgical anastomosis, caustic ingestion, drug-induced esophagitis, and some congenital types of strictures [1,2]. Due mostly to dysphagia, these strictures impair quality of life and can lead to serious adverse effects, such as aspiration, weight loss, and nutritional deficiencies [3]. Therefore, according to the European Society of Gastrointestinal Endoscopy (ESGE) guidelines, upper gastrointestinal endoscopy (UGE) is necessary to visualize the stricture and obtain a biopsy sample to determine the nature of the stricture, whether benign or malignant [4]. The distance between the incisors and the proximal edge, as well as the stricture’s length, diameter, and endoscope passage are all important parameters. Along with image documentation, the existence of ulceration, stricture complexity, and any other anomalies in the remaining esophagus should be noted. Exudates, rings, oedema, furrows, and strictures (EREFS), which are indicative of eosinophilic esophagitis, should be observed but should not be considered as definitive diagnostic criteria replacing biopsies [5].

Strictures are classified from an anatomical standpoint as simple or complex, and based on their response to treatment, as recurrent or refractory. An esophageal stricture (ES) is considered simple if its lumen diameter is larger than 12 mm and its length is less than 20 mm. Simple strictures are easily navigable by an adult endoscope with a 10 mm diameter and are suitable for endoscopic dilatation [6]. It usually takes one to three dilations to alleviate dysphagia symptoms. Simple strictures are most frequently caused by esophageal webs, Schatzki rings, peptic strictures, and eosinophilic esophagitis [7]. Complex esophageal strictures typically exceed 20 mm in length and display irregular, tortuous, or angulated characteristics, accompanied by a significantly narrowed lumen. The luminal diameter is less than 10–12 mm. These strictures may limit the passage of a standard-sized endoscope [6]. Due to their higher recurrence rates, complex strictures present greater management challenges compared to simple ones [8]. The refractory or recurrent benign esophageal strictures (RBESs) are defined as either the inability to maintain a stricture at a diameter of 14 mm after five sessions spaced two weeks apart (refractory) or the failure to sustain a satisfactory luminal diameter for four weeks after achieving 14 mm (recurrent) [9]. Commonly, their etiologic spectrum encompasses post-surgical anastomotic, caustic, radiation strictures, and post-endoscopic therapy such as endoscopic mucosal resection or endoscopic mucosal dissection [10].

The usual treatment for strictures is endoscopic dilatation using balloon or bougie dilators. Balloon dilation is a recognized technique for treating ESs, with reported overall success rates ranging from 67% to 98% in previous studies [11,12,13]. Before dilation, obtaining a tissue diagnosis is essential to distinguish between benign and malignant lesions, as well as to guide the overall management and to assess the risk of perforation [14].

The primary goal of our study was to provide descriptive evidence about the appropriately tailored medical and endoscopic dilation management of BESs in a tertiary endoscopy center from northeastern Romania by assessing the improvement of dysphagia or the need for surgical interventions.

## 2. Materials and Methods

### 2.1. Patients

We performed a retrospective study including patients with difficulty swallowing who underwent UGE and were diagnosed with BESs based on histological assessment from January 2019 to September 2024 at the Gastroenterology and Hepatology Institute, în Iași, Romania.

The inclusion criteria were age ≥ 18 years, signed informed consent, dysphagia based on the Ogilvie score [15], patients who provided assent for UGE evaluation, and those who completed the full endoscopic management program for esophageal strictures (until the endoscopic management was proven to be ineffective or the Ogilvie score reached 0).

**Figure 1:**
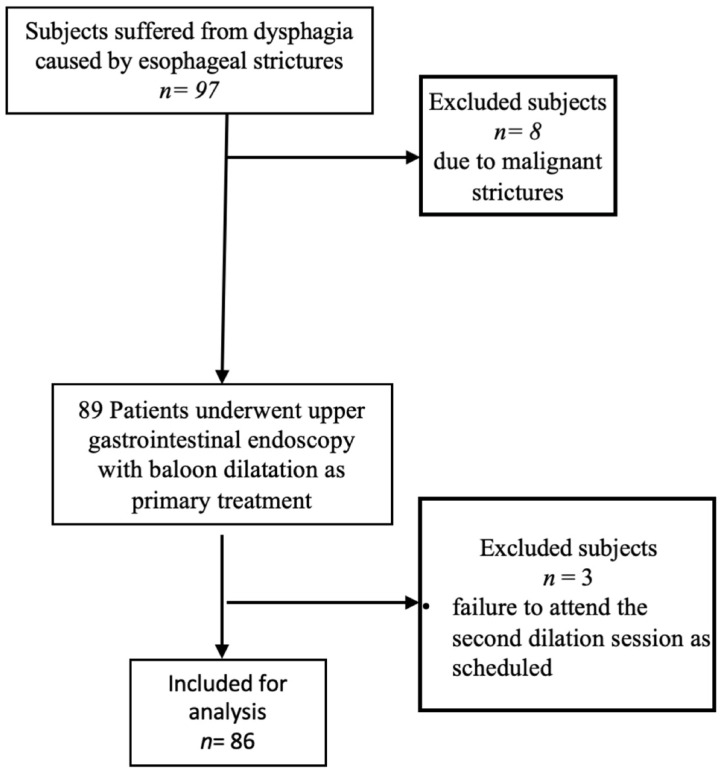
Study flow chart.

Exclusion criteria were the histopathological evidence of malignancy, failure to provide informed consent, and non-attendance at the subsequent dilation session as scheduled (Figure 1). The medical history of the patient, with a particular focus on the evolution of dysphagia, as well as the clinical examination and biochemical profile (complete blood count, coagulation profile, electrolytes, and kidney function) were documented for each patient. Moreover, each stricture was characterized by clinical, endoscopic, histopathological, and imaging features, identifying the underlying nature and etiology of the stricture, such as peptic injury, eosinophilic esophagitis, infectious esophagitis, corrosive damage, congenital abnormalities, iatrogenic factors, and autoimmune conditions. Strictures with no clearly identified etiology have been classified as idiopathic.

### 2.2. Assessment of Esophageal Strictures

All endoscopic procedures were performed under the supervision of an intensive care physician using analgo-sedation (propofol, ketamine, midazolam, and lidocaine). The initial evaluation focused on assessing the stricture’s diameter, length, and classification (simple or complex). For complex strictures which did not allow endoscopic passage (diameter less than 9 mm), further evaluation with a barium swallow or thoracic contrast-enhanced computed tomography (CT) with oral contrast administration was required to accurately visualize the stricture’s morphology. UGE was performed using Fujifilm Eluxeo 7000 (Japan) with a standard 9 mm diameter endoscope. According to Lew RJ et al. the simple strictures are short, straight, and allow the passage of a normal diameter endoscope, and the complex ones are usually longer (≥2 cm), angulated, irregular, or have a severely narrowed diameter. Based on the morphology of the esophageal stricture, we followed the internal dilation protocol, which included the use of Savary-Gillard bougie dilators (Cook Medical, Bloomington, IN, USA), through-the-scope balloon dilators (Controlled Radial Expansion Balloon, Rigiflex, USA), or the placement of fully covered self-expandable metallic stents (SEMS, Cook Medical, Anaheim, CA, USA), for refractory stenoses. This study was conducted in accordance with the principles outlined in the Declaration of Helsinki and received approval from the Ethics Committee of our institution.

### 2.3. Endoscopic Dilatation Session

Firstly, we assessed the degree of dysphagia according to the Ogilvie score. Prior to UGE, patients were instructed to fast for at least 8 h to ensure a clear view of the esophageal lumen and to prevent the risk of aspiration. Antiplatelets and anticoagulants therapies have been managed in accordance with the European Society of Gastrointestinal Endoscopy guidelines [16,17]. Secondly, an imaging workup of the stricture was performed to assess its complexity, followed by the initial endoscopic evaluation for tissue acquisition. Histological samples were used to rule out malignancy. At second endoscopy, simple strictures were managed in the standard endoscopy room, while complex strictures were managed in the fluoroscopy room using a standard digital C-arm machine. The guidewire was placed under endoscopic view through stricture, while a through-the-scope balloon catheter was placed under endoscopic guidance or a Savary-Gillard bougie was passed over the guide wire using fluoroscopy guidance. The time of dilatator pressure to the stricture area was approximately 30 to 60 s, exchanging the dilatator or inflating the balloon with a 3 mm diameter (step-up rule of 3) per dilatation session. Patients were permitted liquid intake 6 h following the procedure and were encouraged to resume semisolid foods the next day unless they experienced complications during the procedure. Subsequently, all patients were closely monitored for any signs or symptoms of esophageal perforation, including chest pain, back pain, shoulder pain, epigastric tenderness, and tachycardia. A chest X-ray was performed after each procedure to check for esophageal perforations, while in patients with endoscopic identification of deep esophageal laceration (defined as follows: superficial laceration—injury associated dilatation limited to mucosa (esophageal tears) and deep laceration—mucosal defect that extends beyond the superficial layers, reaching the submucosa or deeper layers) a CT scan with oral contrast was used to diagnose this complication (7). Patients were initially monitored every two weeks for the first two months. If dysphagia recurred, repeated balloon dilation sessions were conducted every two weeks until symptoms were alleviated. After that, follow-up appointments were scheduled at three-month intervals, and then at six months, to check for any recurrence of dysphagia. In cases of refractory or recurrent benign strictures, we had the option either to place a fully covered over-the-wire self-expandable metallic stent (fc-SEMS) which is kept in place for up to 3 months or to perform a 15 mm balloon dilatation with an injection of corticosteroid in 4 quadrants at 2 weeks. Technical and clinical success rates, adverse events, stricture patency, and need for additional therapy have been evaluated.

### 2.4. Statistical Analysis

The data were processed using IBM SPSS, Version 22.0 (IBM SPSS Inc., Chicago, IL, USA). For categorical variables, statistics are presented as frequencies (percentages). Baseline characteristics and clinical variables are reported as mean ± standard deviation if the data follow a normal distribution, or as median (25th and 75th percentiles) if the data are not normally distributed. The Kolmogorov–Smirnov test was used to assess the distribution. To compare continuous variables between groups with normally distributed data, an unpaired t-test was applied, while the Mann–Whitney or Kruskal–Wallis tests were used for skewed data. Fisher exact test and one-way ANOVA was used to examine differences in factors according to different anatomical esophageal strictures, while the Pearson correlation coefficient (r) was used to assess the relationship between two variables. A Kaplan–Meier survival analysis was performed to evaluate the time-to-event data regarding the need for esophageal dilations in patients with esophageal strictures stratified by etiology. A *p*-value of < 0.05 (two-tailed) was considered statistically significant. Only complete datasets were included in the analysis.

## 3. Results

In our study, we enrolled a total of 86 patients with BESs, more than half of them were males (59.3%) and the mean age was 59.6 ± 14.65 years. In addition, we identified some important comorbidities, such as malnutrition (90.3%), cardiovascular disease (31.4%), neurological diseases (10.5%), type 2 diabetes (8.1%), and metabolic syndrome (3.5%). The anticoagulant therapy was assessed in 5.8% of the patients. The etiological spectrum of the BESs included peptic injury (44.2%), post-caustic (48.8%), post-surgical interventions (4.7%), and Schatzki rings and epidermolysis bullosa (2.2%); 12.8% of the stenosis identified in our department allowed endoscopic passage. All patients underwent dilation with Savary-Gillard bougie dilators (76.7%), pneumatic balloon dilators (23.3%), or both (24.4%), and only one case required the placement of a metallic stent. We also observed some post-procedural complications, such as perforation (2.3%), bleeding (4.7%), and superficial laceration (4.7%). Most of these complications (88.9%) were treated endoscopically, with percutaneous endoscopic gastrostomy (PEG) placement required in only 5.8% of the cases (Table 1)

The location of the strictures was classified into upper third (8.1%), middle third (32.6%), and lower third (59.3%). The average length value was higher in the case of upper strictures (42.5 mm). Our results suggest that the number of dilation sessions varies according to the locations of the stenosis. Therefore, in the upper third the mean number of dilations was 1.5 per patient; in the lower strictures the mean number was 2.5 per patient; and in the middle esophagus strictures the mean number of dilations was 3.5 per patient. The dysphagia score at the end of the follow-up period was different depending on the location of the stenosis; for middle strictures a best mean score of 1 was found, and only 2% of these patients had worsening dysphagia after the procedures compared to those with lower strictures, who exhibit a higher percentage of symptom worsening (4%). Moreover, the need for PEG placement or for esophagectomy was higher in patients with middle third (2%) or lower strictures (3%) compared to those with upper strictures (Table 2).

**Figure 2:**
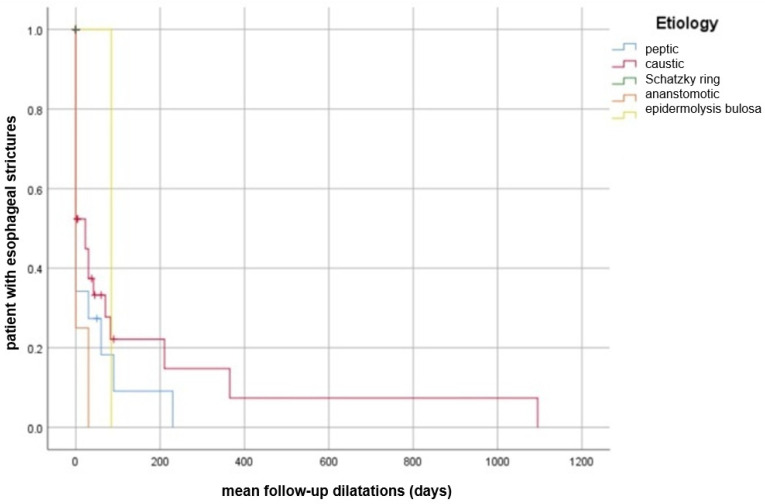
Kaplan–Meier curve demonstrating mean time from first to last endoscopic dilation (ED) session during mean follow-up dilatation sessions of patients with benign esophageal strictures associated with higher number of ED sessions, stratified by etiology.

The majority of the strictures (80.2%) were complex, requiring advanced techniques for management. Endoscopic dilation was the primary treatment, using Savary-Gillard bougie dilators (76.7%) and pneumatic balloon dilators (23.3%). During the follow-up period, patients with post-caustic strictures needed repeated dilatation procedures, with the last procedure performed 2–3 years after the first one; compared to those with peptic stenosis who required the last dilatation after 200 days. On the other hand, patients with anastomotic strictures or Schatzki rings and epidermolysis bullosa required the last dilatation procedures in a mean time of seven days, which means a reduced number of dilatations in contrast with peptic and caustic stenosis (Figure 2).

Clinical success was achieved in 95.3% of patients, with a significant improvement in the Ogilvie dysphagia score. Patients with post-caustic strictures required repeated dilations over the years, compared to shorter intervals for peptic strictures. Refractory strictures (16.3%) required advanced interventions, including fully covered self-expandable metallic stents (fc-SEMS) and corticosteroid injections. Individualized treatment approaches were crucial for these cases. Steroid injections have been used in 15 patients as a rescue resource. No further endoscopic therapy has been performed after the steroid injection. Patients were followed up at two-week intervals after injection and only one patient showed dysphagia remission. All patients were referred for surgical management but only four consented and benefited from surgery. The rest opted for long-term fluid enteral nutrition.

## 4. Discussion

The endoscopic treatment of esophageal strictures has demonstrated a high effectiveness, with clinical success rates typically exceeding 80% for uncomplicated strictures [18] and about 60% in the case of RBESs [19].

Both balloon and bougie dilators can be effectively used for esophageal dilation, with the choice of dilator often being guided by the operator’s experience and the specific characteristics of the stricture [20]. The individualization of the treatment is crucial; the ideal diameter for dilation varies based on the size, etiology, and location of the stricture. For instance, larger diameters may be preferred for post-surgical strictures, while smaller diameters are typically used for proximal strictures [4,6,12]. This tailored approach aligns with guidelines that recommend customizing therapy based on the specific characteristics of the stenosis [21].

In our cohort, similar to previous studies, some patients were managed using multiple types of dilation. However, it is important to note that, unlike bougie dilators, balloon dilators exert radial strain only along the length of the stricture, generating circumferential pressure through internal pressure and diameter, a phenomenon known as hoop stress. Larger balloons with higher radial force require less pressure to expand but come with an increased risk of perforation. The surface area of the stricture significantly influences the dilating force, with longer strictures generally resulting in more effective dilation [22].

Following dilation, patients typically require additional treatments every week or two until both symptomatic improvement and the ability to pass a dilator of at least 15 mm are achieved [23]. Generally, one to three treatment sessions are sufficient to alleviate dysphagia in uncomplicated strictures, with over 95% of patients needing no more than five dilatations [24]. However, there are situations where the symptoms persist after a minimum of five dilation sessions to at least 14 mm, performed every two weeks; these cases are known as RBESs [25]. In these cases, management faces challenges due to the limited effectiveness of standard dilation procedures, complicating the development of standardized protocols [12].

A recent retrospective study has identified some predictive factors for RBESs, including caustic etiology, age under 50 years, the presence of multiple strictures, and a stricture length ≥20 mm [26]. According to the current studies, the etiology of strictures plays a significant role in management strategies, with caustic strictures often necessitating more prolonged and repeated interventions compared to peptic strictures [27]. In our study, caustic stenoses were the most frequent etiology for RBESs, accounting for 71.4% of cases. This result suggests that the etiology of strictures significantly influences management strategies, necessitating a tailored approach for effective treatment.

Several treatment options have been proposed for RBESs to avoid surgical intervention or lifelong PEG placement. Steroid injection has been suggested to enhance the efficacy of dilation procedures [2]. A randomized controlled trial (30 patients) indicated that steroid injection, combined with acid suppression, reduced the number of dilations required and improved the time to subsequent dilation sessions [28]. Such an approach differed from our strategy where steroid injections have been performed after the failure of multiple endoscopic dilation sessions. Thus, similarly to our findings, another study involving a larger cohort (60 patients with anastomotic esophageal strictures) showed no benefits from steroid injection prior to dilation [29]. In our cohort, the patients did not benefit from steroid injections, but they were all on a proton pump inhibitor treatment before and after the procedure, which potentially contributed to the maintenance of the long-term tolerance for fluid enteral nutrition despite the persistent dysphagia.

The primary limitations of this study are its single-center data and retrospective design. The recall bias increases when the stricture dilations that were chosen as the first dilations at our institution are included in the study. However, for the first two months, we monitored each patient every two weeks for additional balloon dilatation if needed, according to our University Hospital internal protocol.

In addition to traditional dilation methods, self-expandable plastic or metal stents (SEPSs/SEMSs) and at-home self-dilation have been suggested for patients with RBESs. However, the effectiveness of these therapies remains uncertain due to short-term follow-up and inconsistent results [30].

**Figure 3:**
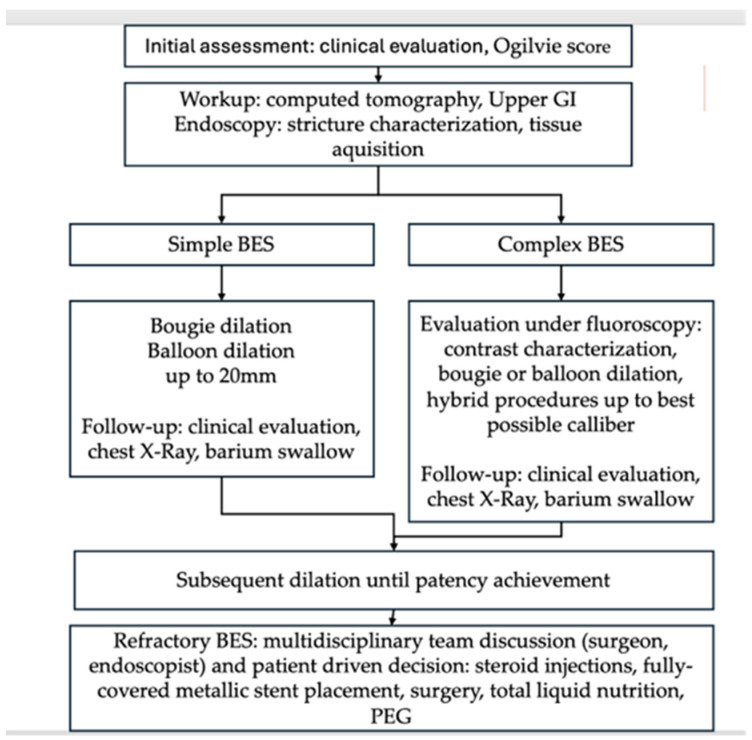
Strategy for tailored management of benign esophageal strictures (BESs) guided by current findings and literature assessment. PEG: percutaneous endoscopic gastrostomy.

The most frequently reported post-procedural complications in recent studies include bleeding, pulmonary aspiration, and perforation [17]. In our patient group, adverse events were rare, with perforation occurring in 2.3%, superficial lacerations in 4.7%, and bleeding in 4.7%. Most of the patients experienced favorable post-procedural outcomes (95.3%). Although concerning events like perforation and bleeding were rare, they were effectively managed through endoscopic techniques (only one case required surgical intervention). The strategy shown in Figure 3 highlights a potential patient-centered approach to benign esophageal strictures based on our findings and the current literature.

## 5. Conclusions

This study underscores the importance of individualized management strategies and highlights the effectiveness of endoscopic dilation in improving patient outcomes for BESs. Regular follow-ups and tailored treatment protocols, based on stricture complexity and location, are essential to address recurrence and refine treatment protocols. While adverse events like perforation and bleeding were rare, they were effectively managed predominantly through endoscopic measures. Importantly, the etiology of strictures influenced management strategies, with caustic strictures necessitating more prolonged and repeated interventions compared to peptic strictures. More evidence-based data are needed, especially regarding the use of advanced techniques like corticosteroid injections or stenting for refractory cases.

## Figures and Tables

**Table 1:** Clinical and endoscopic characteristics of patients.

Demographics and Clinic Characteristics Baseline	Total of 86 Patients
Age, mean (SD)	59.66 (14.65)
Male, *n* (%)	51 (59.3)
Duration from first diagnosis (follow-up period)	
>5 years, *n* (%)	37 (43)
2–5 years	21 (24.5)
<2 years	28 (32.5)
Total Dysphagia, *n* (%)	64 (74.4)
Ogilvie score, mean (SD)	3.73 (0.45)
Intervals from caustic ingestion to diagnosis, (months)	14.87 (12)
Etiology	
Peptic strictures, *n* (%)	38 (44.2)
Anastomotic strictures, *n* (%)	4 (4.7)
Caustic strictures, *n* (%)	42 (48.8)
Schatzki ring, *n* (%)	1 (1.2)
Epidermolysis bullosa, *n* (%)	1 (1.2)
Stricture passed with the endoscope, *n* (%)	11 (12.8)
Type of stricture	
Simple, *n* (%)	17 (19.8)
Complex, *n* (%)	69 (80.2)
Recurrent stricture, *n* (%)	12 (14)
Refractory stricture, *n* (%)	14 (16.3)
Location of proximal part of stricture, *n* (%)	
Upper (18–23 cm), *n* (%)	7 (8.1)
Middle (24–31 cm), *n* (%)	28 (32.6)
Lower (32–40 cm), *n* (%)	51 (59.3)
Length of stenosis, mean (SD)	36.37 (26.65)
Endoscopic management	
Mean number of dilatations, (SD)	3.5 (2.5)
Mean interval between dilatations 1 and 2, (longest interval), days	209.8 (461)
Mean interval between dilatations 2 and 3, (longest interval), days	116 (266.4)
Mean intervals of dilatations > 3 dilatations, (longest interval), days	67.71 (208.28)
Initial diameter of stricture, median (IQR)	9 (3–18)
Final diameter of stricture, median (IQR)	12 (9–20)
Ogilvie score, mean (SD) at end of procedures	1.34 (1)
Instruments	
Savary-Gillard, *n* (%)	66 (76.7)
Balloon, *n* (%)	20 (23.3)
Savary + Balloon, *n* (%)	21 (24.4)
FC-SEMS, *n* (%)	1 (1.2)
Complications	
Perforation, *n* (%)	2 (2.3)
Superficial laceration, *n* (%)	4 (4.7)
Bleeding, *n* (%)	4 (4.7)
Complications management	
Endoscopy, *n* (%)	8 (88.9)
Surgical, *n* (%)	1 (11.1)
Favorable post-procedural evolution, *n* (%)	82 (95.3)
Requirement of PEG, *n* (%)	5 (5.8)
Comorbidities	
Neurologic, *n* (%)	9 (10.5)
Malnutrition, *n* (%)	80 (90.3)
Metabolic syndrome, *n* (%)	3 (3.5)
Type 2 diabetes mellitus, *n* (%)	7 (8.1)
Cardiovascular disease, *n* (%)	27 (31.4)
Anticoagulants, *n* (%)	5 (5.8)

**Table 2:** Overall outcomes of stricture dilation.

Stricture Length at Study Inclusion	Upper 18–23 cm (7 Patients)	Middle 24–31 cm (28 Patients)	Lower 32–40 cm (51 Patients)	*p*
Age, mean (SD)	48.67 (9.65)	52.71 (13.38)	56.92 (15.64)	0.015
Intervals of ingestion, months	23.75 (15.54)	14.21 (13.42)	12.67 (8.65)	
Stricture diameter, mm, median (IQR)	8 (7–11)	8 (3–14)	5.5 (5–11)	0.092
Length of stenosis, median (IQR)	42.5 (20–80)	35 (10–100)	25 (10–30)	0.009
No. of dilations, median (IQR)	1.5 (1–7)	3.5 (1–8)	2.5 (1–11)	0.371
No. of >3 dilations/days follow-up, mean (SD)	160.71 (412)	20.66 (28.52)	53.92 (114.85)	0.026
Dysphagia score at study inclusion, median (IQR)	4 (3–4)	3 (2–4)	4 (1–4)	0.165
Dysphagia score at end of follow-up, median (IQR)	1.5 (1–3)	1	1.5 (1–3)	0.259
Unchanged or worse dysphagia at end of follow-up	0	0	4	0.001
Need for PEG, no. (%)	0	2	3	0.001
Need for esophagectomy, no. (%)	0	0	4	0.001

## Data Availability

Supporting data are available upon request from the corresponding authors.
